# 

**Published:** 1888-02

**Authors:** 


					﻿Vol. IX.
i- LBKUAKY. 1 odo.
NO. 2.
arrears
iWMwnuraiiwr:
JL M01THXY J OTO NAIL
DEVOTED TO
DENTAL AND ORAL SCIENCE.
W. C. BARRETT, M. D., D. D. S.,
EDITOR,.
The Iew York Dental Journal Association,
PUBLISHERS,
No. 35 West <L6tli Street, New York,
AND
No. 308 Franklin St., Buffalo. N.Y.
$2.50 Per Annum in Advance, .	... Single Copies, 25 Cents.
Entered at the Post-Office, Buffalo, N. Y., as Second-Class matter.
				

